# Synthesis and structure of poly[(μ_3_-hydrogen phosphato)(pyridine)­zinc(II)]

**DOI:** 10.1107/S2056989025008308

**Published:** 2025-10-24

**Authors:** Liming Huang, Fochang Luo, Qingshi Wang, Xian Xu, Zhifeng Pan

**Affiliations:** a Orthopedic Department, The Second Hospital of Sanming, Fujian, 366000, People’s Republic of China; b Clinical Laboratory, Orthopedic Department, The Second Hospital of Sanming, Fujian, 366000, People’s Republic of China; c Medical Department, The Second Hospital of Sanming, Fujian, 366000, People’s Republic of China; University of Missouri-Columbia, USA

**Keywords:** zinc, metal phosphate, hydrogen bond, supra­molecular structure, crystal structure

## Abstract

The title compound, [Zn(HPO_4_)(C_5_H_5_N)]_*n*_ or pyZn(HPO_4_) (**1**), was prepared from the solvothermal reaction of [Mo_3_O_2_(O_2_CCH_3_)_6_(H_2_O)_3_]ZnCl_4_ and H_3_PO_4_ in a mixture of pyridine and water. It displays an infinite ladder structure built of alternately arranged ZnO_3_N and PO_3_(OH) tetra­hedra, linked O—H⋯O hydrogen bonds into supra­molecular sheets. C—H⋯O inter­actions between CH groups of the pyridine rings and phosphate groups connect the sheets into a three-dimensional framework structure.

## Abstract 2

The title compound displays an infinite ladder structure built of alternately arranged ZnO_3_N and PO_3_(OH) tetra­hedra, linked O—H⋯O hydrogen bonds into supra­molecular sheets. C—H⋯O inter­actions between CH groups of the pyridine rings and phosphate groups connect the sheets into a three-dimensional framework structure.

## 1. Chemical context

Divalent metal phosphates such as hy­droxy­lapatite [Ca_5_(PO_4_)_3_(OH)], the main component of human bones, play an essential role in body structure. Zinc phosphates are extensively involved in bone development, dental materials, environmentally friendly anti­corrosive and anti­rust pigments and industrial additives. They exhibit a vast structural diversity including cluster, chain, layer and open-framework structures (Mao *et al.*, 2020 ▸; Amghouz *et al.*, 2014 ▸; Chen *et al.*, 2007 ▸; Lin *et al.*, 2003*b* ▸, 2007 ▸; Yang *et al.*, 2009 ▸; Choudhury *et al.*, 2000 ▸; Rayes *et al.*, 2001 ▸). A number of *L*Zn(H*_x_*PO_4_) where Zn^2+^ is datively coordinated to Lewis basic ligands [*x *= 0–2; *L* = Cl^−^ (Chen *et al*., 2007 ▸; Rayes *et al*., 2001 ▸); NH_3_ (Amghouz *et al*., 2014 ▸); 5-(4-pyrid­yl)tetra­zolate) (Yang *et al*., 2009 ▸); 1,10-phenanthroline (Lin *et al.*, 2003*a* ▸); 4,4 -dimethyl-2,2-dipyridyl, 5,5-dimethyl-2,2-dipyridly (Lin *et al*., 2007 ▸) 1,2-di­methyl­imidazole (Mao *et al*., 2020 ▸); 4*H*-1,2,4-triazole-κ*N*^1^ (Aitenneite *et al*., 2012 ▸); CaZn_2_Fe(PO_4_)_3_ (Khmiyas *et al.*, 2016 ▸)] to form discrete or one-dimensional ladder structures. Herein, a new family member of zinc phosphates datively coordinated by an aromatic pyridine ligand, namely pyZn(HPO_4_) (**1**), is reported including its synthesis, isolation and single-crystal structural characterization.

## 2. Structural commentary

**Figure 1:**
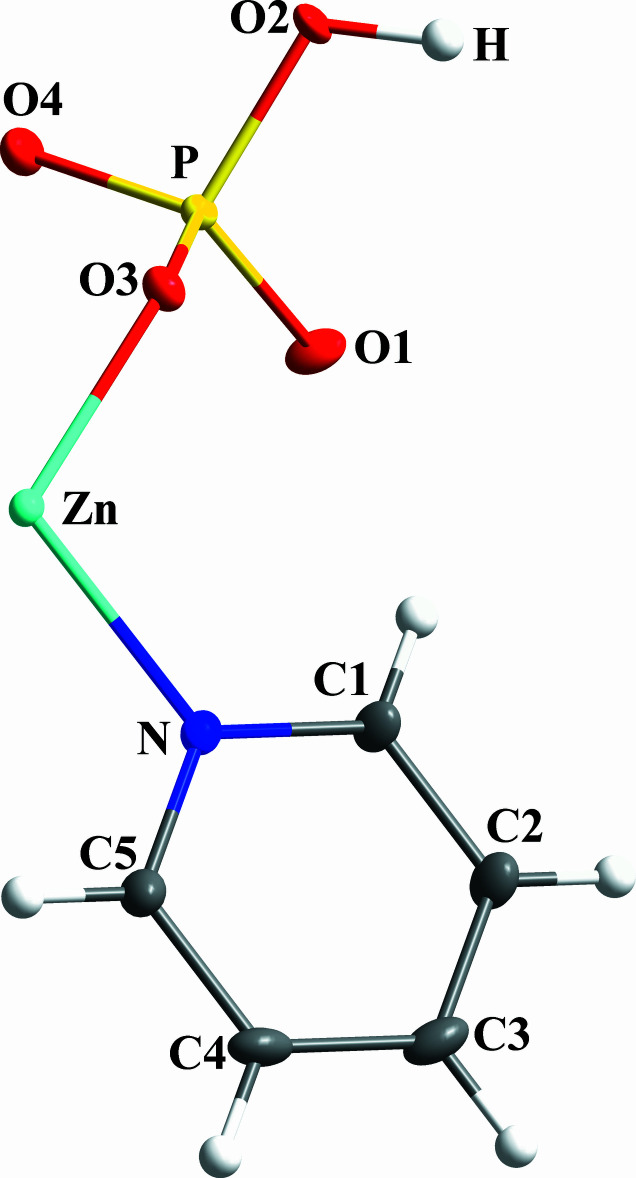
The asymmetric unit of **1** with 50% probability displacement ellipsoids.

**Figure 2:**
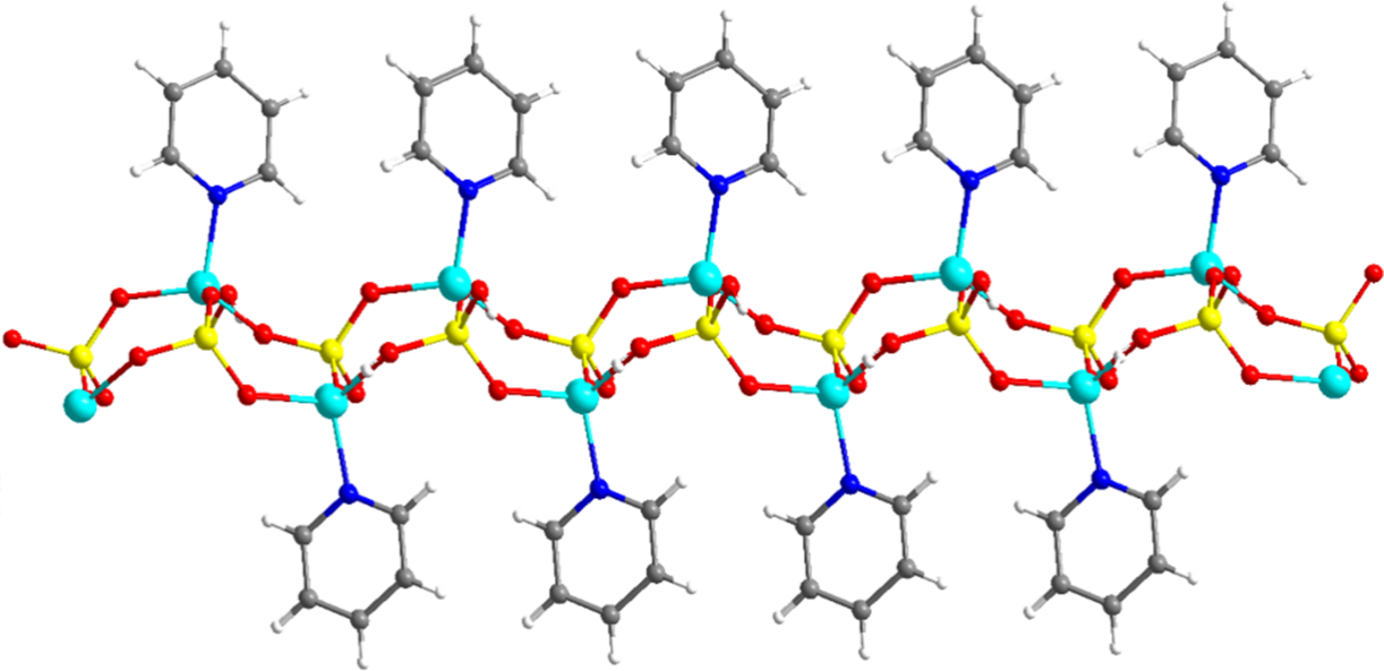
One-dimensional ladder structure of **1**.

As illustrated in Fig. 1 ▸, the asymmetric unit of the title compound, [pyZn(HPO_4_)]_*n*_, contains one Zn^2+^ cation, one (HPO_4_)^2−^ anion and an pyridine ligand. The Zn^2+^ cation is coordinated by three O atoms from three phosphate ligands and the Lewis basic N atom from the pyridine ligand in a quite regular tetra­hedral geometry with bond angles in the range 110.2 (1)–116.6 (1)°. Each phosphate anion is connected to three Zn^2+^ cations with the strict alternation of ZnO_3_N tetra­hedra and HPO_4_ tetra­hedra giving rise to an extended ladder structure (Fig. 2 ▸) characteristic of Zn_2_P_2_O_4_ eight-membered rings. The Zn—O bonds range from 1.911 (2) to 1.941 (3) Å, similar to the reported values, but at 2.043 (3) Å the Zn—N bond is markedly longer than those for example in Zn-mmim [1,2-di­methyl­imidazole, 1.988 (2) Å; Mao *et al.* 2020 ▸)], indicative of weaker Zn–py bonding. The P—O bond lengths fall in the range 1.505 (3)–1.579 (3) Å, similar to those [1.508 (2)–1.587 (2) Å] in mmimZnHPO_4_ (Mao *et al.* 2020 ▸) with the longest P—O bond being assigned to the P—OH group.
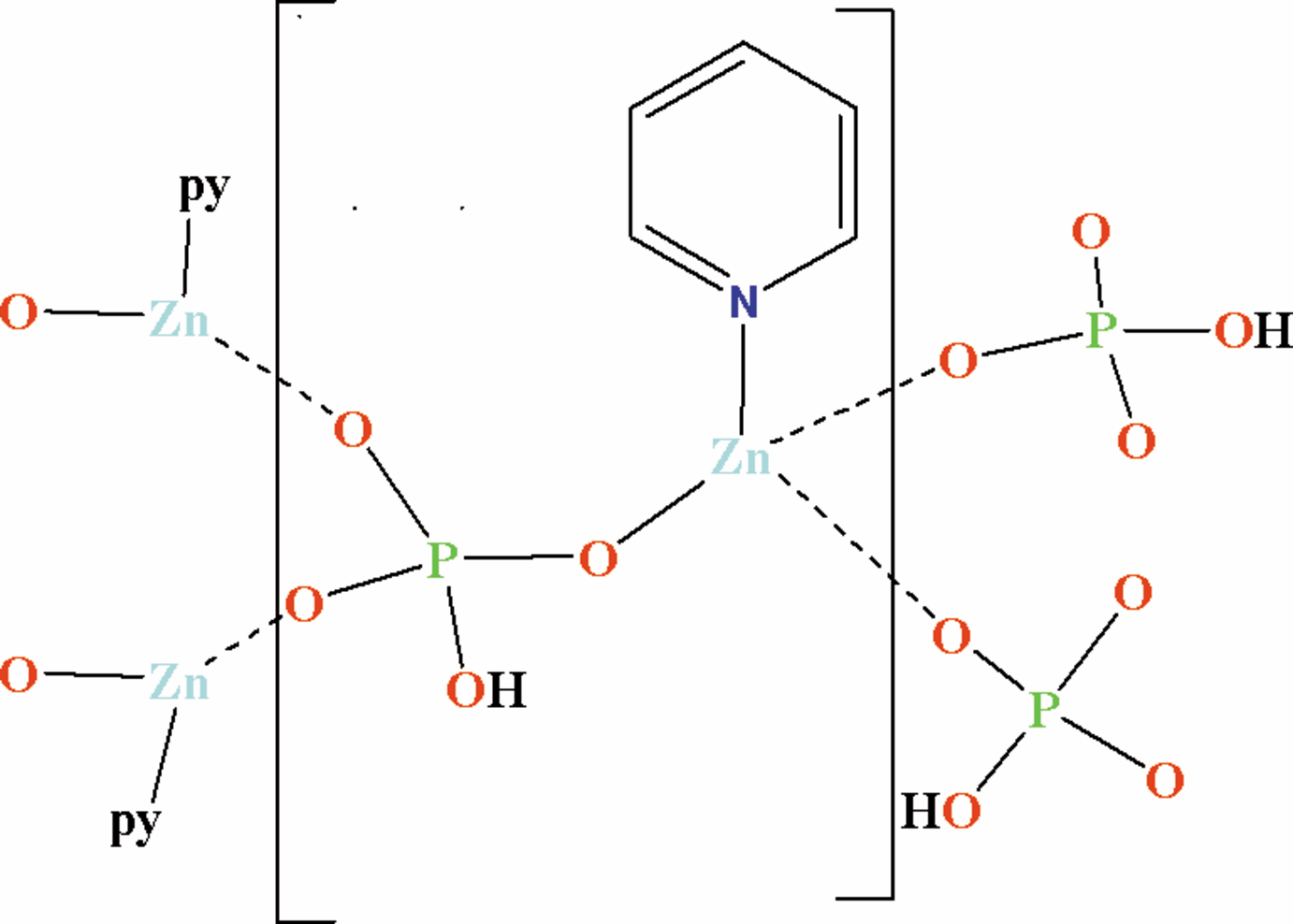


## 3. Supra­molecular features

**Figure 3:**
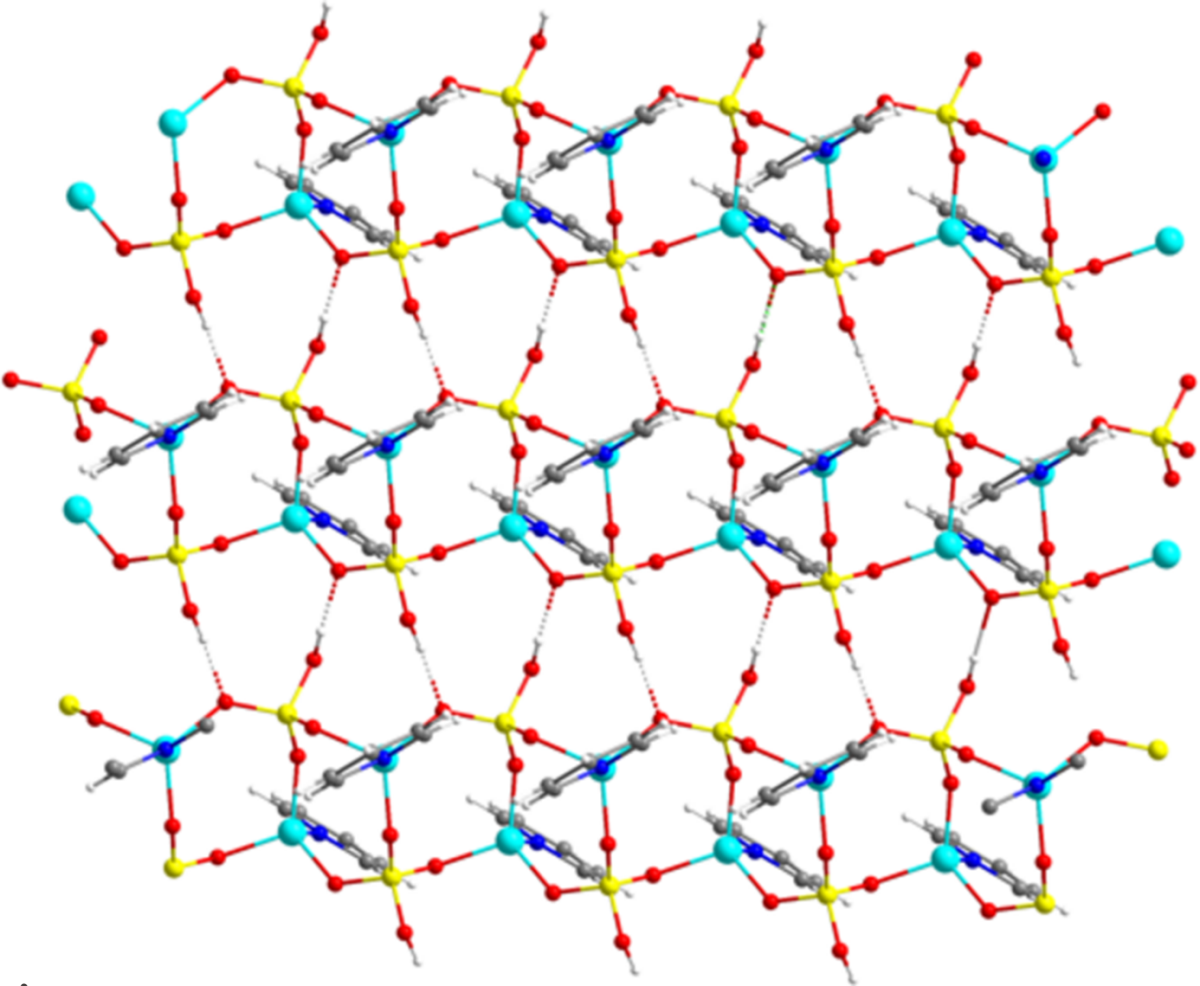
Two-dimensional hydrogen-bonded structure of **1**.

**Figure 4:**
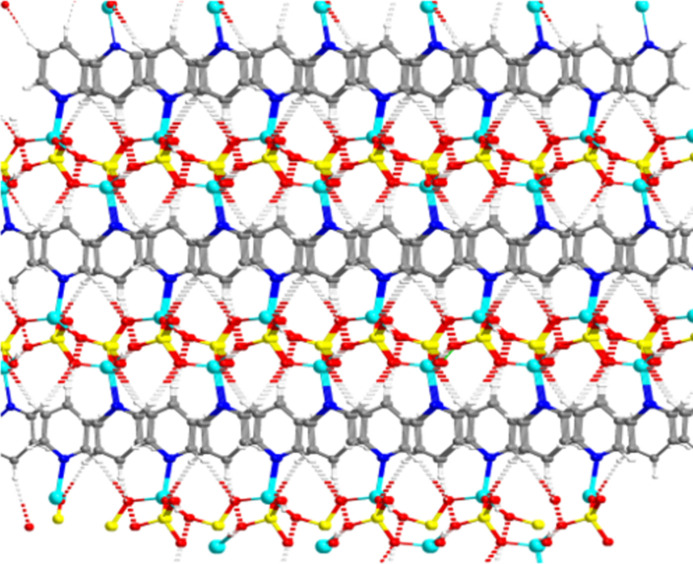
Three-dimensional hydrogen-bonded framework of **1**.

Fig. 3 ▸ illustrates the hydrogen-bonded sheet of **1** (numerical details of the hydrogen bonds are given in Table 1 ▸). The short OH⋯O separation of 2.647 (3) Å and almost linear O—H⋯O angle [168 (4)°] indicate the significant hydrogen bonding inter­actions between the ladders. The hydrogen bonding network are characteristic of the P_2_ZnO_5_H_2_ ten-membered ring as demonstrated in Fig. 3 ▸. The pyridine ligands are almost perpendicular to the hydrogen binding layers. There are significant inter­actions between the hydrogen-bonded sheets through C—H⋯O—P inter­actions (Table 1 ▸), which lead to the formation of three-dimensional supra­molecular framework as shown in Fig. 4 ▸.

## 4. Database survey

A Cambridge Structural Database online search (July 17, 2025; Groom *et al.*, 2016 ▸) for the [(*μ*_3_-hydrogen­phosphato)(pyridine)­zinc] unit yielded no hits, indicating that no zinc phosphates coordinated by pyridine ligands have been reported. A search for zinc phosphates datively coordinated by N-donor ligands revealed several species containing NH_3_ (Amghouz *et al.*, 2014 ▸); 5-(4-pyrid­yl)tetra­zolate) (Yang *et al.*, 2009 ▸); 1,10-phenanthroline (Lin *et al.*, 2003*a* ▸); 4,4-dimethyl-2,2 -dipyridyl, 5,5-dimethyl-2,2-dipyridyl (Lin *et al.*, 2007 ▸) 1,2-di­methyl­imidazole (Mao *et al.*, 2020 ▸); 4H-1,2,4-triazole-*kN*^1^ (Aitenneite *et al.*, 2012 ▸).

## 5. Synthesis and crystallization

**Figure 5:**
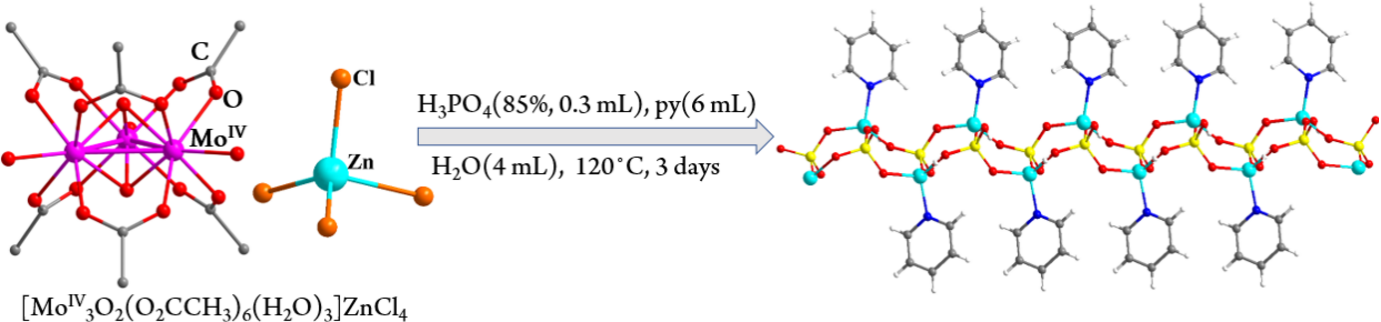
Solvothermal synthesis of **1**.

[Mo_3_O_2_(O_2_CCH_3_)_6_(H_2_O)_3_]ZnCl_4_·8H_2_O (0.1 g, 0.1 mmol) (Xu *et al.*, 2018 ▸, 2025 ▸) was added to a mixture of H_3_PO_4_ (85%, 0.3 ml), pyridine (py, 6 ml) and water (4 ml). The resulting mixture was sealed in a 25 ml Teflon-lined steel autoclave and heated at 393 K for three days. The reactor was cooled to room temperature at a rate of 4 K h^−1^ to produce colourless crystals of pyZn(HPO_4_) (**1**), differing from the previous synthetic methodology of zinc phosphates wherein zinc sources were from zinc oxides or zinc salts such as Zn(O_2_CCH_3_)_2_. The synthesis is shown in Fig. 5 ▸. Notably, the solvothermal reactions using zinc oxides or zinc salts instead of [Mo_3_O_2_(O_2_CCH_3_)_6_(H_2_O)_3_]ZnCl_4_ failed to produce pyZn(HPO_4_) (**1**), indicative of some role of the dianionic group ZnCl_4_^2−^ as zinc source.

## 6. Refinement

Crystal data, data collection and structure refinement details are summarized in Table 2 ▸. The crystal studied was refined as an inversion twin.

## Supplementary Material

Crystal structure: contains datablock(s) I. DOI: 10.1107/S2056989025008308/ev2021sup1.cif

Structure factors: contains datablock(s) I. DOI: 10.1107/S2056989025008308/ev2021Isup2.hkl

CCDC reference: 2472401

Additional supporting information:  crystallographic information; 3D view; checkCIF report

## Figures and Tables

**Table 1:** Hydrogen-bond geometry (Å, °)

*D*—H⋯*A*	*D*—H	H⋯*A*	*D*⋯*A*	*D*—H⋯*A*
O2—H⋯O3^i^	0.94 (4)	1.73 (4)	2.647 (3)	168 (4)
C1—H1⋯O1^ii^	0.98 (4)	2.58 (4)	3.452 (5)	149 (3)
C3—H3⋯O3^iii^	0.96 (2)	2.61 (3)	3.465 (5)	148 (3)
C4—H4⋯O4^iv^	0.95 (4)	2.49 (4)	3.354 (5)	152 (3)

Symmetry codes: (i) 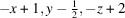; (ii) 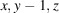; (iii) 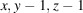; (iv) 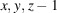.

**Table 2:** Experimental details

Crystal data	
Chemical formula	[Zn(HPO_4_)(C_5_H_5_N)]
*M*_r_	240.45
Crystal system, space group	Monoclinic, *P*2_1_
Temperature (K)	150
*a*, *b*, *c* (Å)	7.7394 (3), 5.3806 (2), 9.0929 (4)
β (°)	91.246 (2)
*V* (Å^3^)	378.56 (3)
*Z*	2
Radiation type	Mo *K*α
μ (mm^−1^)	3.42
Crystal size (mm)	0.21 × 0.15 × 0.06

Data collection	
Diffractometer	Bruker APEXII CCD
Absorption correction	Multi-scan (*SADABS*; Krause *et al.*, 2015 ▸)
*T*_min_, *T*_max_	0.789, 1.000
No. of measured, independent and observed [*I* > 2σ(*I*)] reflections	2993, 1489, 1467
*R*_int_	0.032
(sin θ/λ)_max_ (Å^−1^)	0.649

Refinement	
*R*[*F*^2^ > 2σ(*F*^2^)], *wR*(*F*^2^), *S*	0.023, 0.066, 0.87
No. of reflections	1489
No. of parameters	134
No. of restraints	7
H-atom treatment	All H-atom parameters refined
Δρ_max_, Δρ_min_ (e Å^−3^)	0.48, −0.42
Absolute structure	Refined as an inversion twin
Absolute structure parameter	0.051 (19)

Computer programs: *APEX2* and *SAINT* (Bruker, 2014 ▸), *SHELXT2014/5* (Sheldrick, 2015*a* ▸), *SHELXL2016/6* (Sheldrick, 2015*b* ▸) and *DIAMOND* (Brandenburg, 2005 ▸).

## supplementary crystallographic information

### Poly[(µ3-hydrogen phosphato)(pyridine)zinc(II)] Crystal data

**Table:**

[Zn(HPO_4_)(C_5_H_5_N)]	*F*(000) = 240
*M**_r_* = 240.45	*D*_x_ = 2.109 Mg m^−^^3^
Monoclinic, *P*2_1_	Mo *K*α radiation, λ = 0.71073 Å
*a* = 7.7394 (3) Å	Cell parameters from 2993 reflections
*b* = 5.3806 (2) Å	θ = 3.5–27.5°
*c* = 9.0929 (4) Å	µ = 3.42 mm^−^^1^
β = 91.246 (2)°	*T* = 150 K
*V* = 378.56 (3) Å^3^	Plate, white
*Z* = 2	0.21 × 0.15 × 0.06 mm

### Poly[(µ3-hydrogen phosphato)(pyridine)zinc(II)] Data collection

**Table:**

Bruker APEXII CCD diffractometer	1467 reflections with *I* > 2σ(*I*)
φ and ω scans	*R*_int_ = 0.032
Absorption correction: multi-scan (SADABS; Krause *et al.*, 2015)	θ_max_ = 27.5°, θ_min_ = 3.5°
*T*_min_ = 0.789, *T*_max_ = 1.000	*h* = −10→10
2993 measured reflections	*k* = −6→6
1489 independent reflections	*l* = −10→11

### Poly[(µ3-hydrogen phosphato)(pyridine)zinc(II)] Refinement

**Table:**

Refinement on *F*^2^	Hydrogen site location: difference Fourier map
Least-squares matrix: full	All H-atom parameters refined
*R*[*F*^2^ > 2σ(*F*^2^)] = 0.023	*w* = 1/[σ^2^(*F*_o_^2^) + (0.0516*P*)^2^] where *P* = (*F*_o_^2^ + 2*F*_c_^2^)/3
*wR*(*F*^2^) = 0.066	(Δ/σ)_max_ = 0.001
*S* = 0.87	Δρ_max_ = 0.48 e Å^−^^3^
1489 reflections	Δρ_min_ = −0.42 e Å^−^^3^
134 parameters	Absolute structure: Refined as an inversion twin
7 restraints	Absolute structure parameter: 0.051 (19)

### Poly[(µ3-hydrogen phosphato)(pyridine)zinc(II)] Special details

**Table:**

Geometry. All esds (except the esd in the dihedral angle between two l.s. planes) are estimated using the full covariance matrix. The cell esds are taken into account individually in the estimation of esds in distances, angles and torsion angles; correlations between esds in cell parameters are only used when they are defined by crystal symmetry. An approximate (isotropic) treatment of cell esds is used for estimating esds involving l.s. planes.
Refinement. Refined as an inversion twin.

### Poly[(µ3-hydrogen phosphato)(pyridine)zinc(II)] Fractional atomic coordinates and isotropic or equivalent isotropic displacement parameters (Å^2^)

**Table:**

	*x*	*y*	*z*	*U*_iso_*/*U*_eq_	
Zn	0.84495 (4)	0.37175 (6)	0.86294 (3)	0.00820 (15)	
P	0.75242 (9)	0.8661 (2)	1.02007 (8)	0.00782 (19)	
O1	0.7596 (4)	0.7061 (6)	0.8821 (3)	0.0141 (5)	
O2	0.5993 (3)	0.7752 (6)	1.1188 (3)	0.0133 (5)	
H	0.496 (5)	0.724 (13)	1.073 (5)	0.041 (17)*	
O3	0.7118 (3)	1.1366 (5)	0.9761 (3)	0.0105 (5)	
O4	0.9091 (3)	0.8453 (7)	1.1202 (3)	0.0150 (6)	
N	0.7918 (4)	0.3106 (6)	0.6448 (3)	0.0108 (7)	
C1	0.6998 (5)	0.1149 (8)	0.5928 (4)	0.0134 (7)	
H1	0.670 (5)	−0.016 (7)	0.662 (4)	0.010 (12)*	
C2	0.6540 (5)	0.0929 (8)	0.4445 (4)	0.0178 (8)	
H2	0.589 (7)	−0.050 (8)	0.416 (5)	0.035 (15)*	
C3	0.7034 (6)	0.2769 (8)	0.3478 (4)	0.0176 (8)	
H3	0.667 (5)	0.283 (9)	0.246 (3)	0.014 (12)*	
C4	0.8002 (5)	0.4766 (8)	0.4008 (4)	0.0172 (8)	
H4	0.831 (6)	0.623 (7)	0.349 (5)	0.021 (14)*	
C5	0.8404 (5)	0.4859 (8)	0.5486 (4)	0.0150 (8)	
H5	0.894 (6)	0.623 (7)	0.594 (5)	0.017 (12)*	

### Poly[(µ3-hydrogen phosphato)(pyridine)zinc(II)] Atomic displacement parameters (Å^2^)

**Table:**

	*U*^11^	*U*^22^	*U*^33^	*U*^12^	*U*^13^	*U*^23^
Zn	0.0081 (2)	0.0080 (2)	0.0085 (2)	0.00058 (18)	−0.00045 (13)	−0.00030 (19)
P	0.0070 (3)	0.0077 (4)	0.0087 (4)	0.0005 (5)	0.0001 (3)	0.0012 (5)
O1	0.0213 (13)	0.0094 (12)	0.0117 (13)	0.0050 (11)	0.0012 (10)	−0.0018 (11)
O2	0.0093 (12)	0.0185 (13)	0.0121 (12)	−0.0043 (11)	0.0003 (9)	0.0040 (11)
O3	0.0098 (11)	0.0081 (12)	0.0136 (12)	0.0004 (10)	0.0014 (9)	0.0000 (10)
O4	0.0091 (10)	0.0213 (16)	0.0144 (11)	−0.0005 (12)	−0.0015 (8)	0.0032 (13)
N	0.0100 (13)	0.0109 (17)	0.0116 (14)	0.0011 (10)	−0.0009 (10)	−0.0007 (11)
C1	0.0172 (18)	0.0097 (16)	0.0132 (17)	−0.0017 (15)	−0.0011 (14)	−0.0020 (14)
C2	0.023 (2)	0.016 (2)	0.0146 (18)	−0.0008 (15)	−0.0040 (16)	−0.0034 (16)
C3	0.022 (2)	0.0205 (18)	0.0107 (17)	0.0075 (16)	0.0001 (15)	−0.0025 (15)
C4	0.0170 (19)	0.020 (2)	0.0145 (17)	0.0021 (16)	0.0037 (15)	0.0030 (16)
C5	0.0152 (19)	0.015 (2)	0.0149 (18)	−0.0035 (15)	0.0010 (15)	−0.0021 (16)

### Poly[(µ3-hydrogen phosphato)(pyridine)zinc(II)] Geometric parameters (Å, º)

**Table:**

Zn—O4^i^	1.911 (2)	N—C1	1.351 (5)
Zn—O1	1.926 (3)	C1—C2	1.392 (5)
Zn—O3^ii^	1.941 (3)	C1—H1	0.97 (2)
Zn—N	2.043 (3)	C2—C3	1.383 (6)
P—O4	1.505 (3)	C2—H2	0.95 (3)
P—O1	1.523 (3)	C3—C4	1.390 (6)
P—O3	1.540 (3)	C3—H3	0.96 (2)
P—O2	1.579 (3)	C4—C5	1.374 (6)
O2—H	0.93 (3)	C4—H4	0.95 (3)
N—C5	1.345 (5)	C5—H5	0.94 (2)
			
O4^i^—Zn—O1	113.89 (14)	C5—N—Zn	117.7 (3)
O4^i^—Zn—O3^ii^	116.63 (13)	C1—N—Zn	124.0 (3)
O1—Zn—O3^ii^	111.95 (11)	N—C1—C2	121.8 (4)
O4^i^—Zn—N	104.18 (11)	N—C1—H1	118 (3)
O1—Zn—N	100.16 (12)	C2—C1—H1	120 (3)
O3^ii^—Zn—N	108.16 (12)	C3—C2—C1	119.1 (4)
O4—P—O1	114.37 (17)	C3—C2—H2	124 (3)
O4—P—O3	112.61 (19)	C1—C2—H2	117 (3)
O1—P—O3	109.36 (16)	C2—C3—C4	119.2 (4)
O4—P—O2	103.77 (15)	C2—C3—H3	124 (3)
O1—P—O2	109.52 (18)	C4—C3—H3	117 (3)
O3—P—O2	106.80 (16)	C5—C4—C3	118.4 (4)
P—O1—Zn	128.43 (18)	C5—C4—H4	114 (3)
P—O2—H	119 (3)	C3—C4—H4	128 (3)
P—O3—Zn^iii^	130.24 (16)	N—C5—C4	123.4 (4)
P—O4—Zn^iv^	146.21 (16)	N—C5—H5	113 (3)
C5—N—C1	118.1 (3)	C4—C5—H5	123 (3)
			
O4—P—O1—Zn	40.3 (3)	C5—N—C1—C2	−0.5 (5)
O3—P—O1—Zn	167.62 (19)	Zn—N—C1—C2	174.2 (3)
O2—P—O1—Zn	−75.7 (2)	N—C1—C2—C3	−0.3 (6)
O4—P—O3—Zn^iii^	64.0 (2)	C1—C2—C3—C4	1.2 (6)
O1—P—O3—Zn^iii^	−64.4 (3)	C2—C3—C4—C5	−1.3 (6)
O2—P—O3—Zn^iii^	177.22 (19)	C1—N—C5—C4	0.5 (6)
O1—P—O4—Zn^iv^	55.2 (5)	Zn—N—C5—C4	−174.6 (3)
O3—P—O4—Zn^iv^	−70.5 (4)	C3—C4—C5—N	0.4 (6)
O2—P—O4—Zn^iv^	174.4 (4)		

Symmetry codes: (i) −*x*+2, *y*−1/2, −*z*+2; (ii) *x*, *y*−1, *z*; (iii) *x*, *y*+1, *z*; (iv) −*x*+2, *y*+1/2, −*z*+2.

### Poly[(µ3-hydrogen phosphato)(pyridine)zinc(II)] Hydrogen-bond geometry (Å, º)

**Table:**

*D*—H···*A*	*D*—H	H···*A*	*D*···*A*	*D*—H···*A*
O2—H···O3^v^	0.94 (4)	1.73 (4)	2.647 (3)	168 (4)
C1—H1···O1^ii^	0.98 (4)	2.58 (4)	3.452 (5)	149 (3)
C3—H3···O3^vi^	0.96 (2)	2.61 (3)	3.465 (5)	148 (3)
C4—H4···O4^vii^	0.95 (4)	2.49 (4)	3.354 (5)	152 (3)

Symmetry codes: (ii) *x*, *y*−1, *z*; (v) −*x*+1, *y*−1/2, −*z*+2; (vi) *x*, *y*−1, *z*−1; (vii) *x*, *y*, *z*−1.
